# Monitoring vaccine and non-vaccine HPV type prevalence in the post-vaccination era in women living in the Basilicata region, Italy

**DOI:** 10.1186/s12879-018-2945-8

**Published:** 2018-01-15

**Authors:** Francesca Carozzi, Donella Puliti, Cristina Ocello, Pasquale Silvio Anastasio, Espedito Antonio Moliterni, Emilia Perinetti, Laurence Serradell, Elena Burroni, Massimo Confortini, Paola Mantellini, Marco Zappa, Géraldine Dominiak-Felden

**Affiliations:** 1 Istituto per lo Studio e la Prevenzione Oncologica (ISPO), Florence, Italy; 2 Ospedale Madonna delle Grazie di Matera, Matera, Italy; 3 Dipartimento di Prevenzione, Azienda Sanitaria Locale di Matera, Matera, Italy; 4 Medical and Scientific Department, Sanofi-Pasteur MSD, Rome, Italy; 5 Franchise Development, Sanofi-Pasteur MSD, 162 avenue Jean-Jaurès, CS 50712 69367, Cedex 07 Lyon, France

**Keywords:** HPV type prevalence, genotyping, quadrivalent HPV vaccine, vaccine effectiveness, cross-protection, type-replacement

## Abstract

**Background:**

A large free-of-charge quadrivalent HPV (qHPV) vaccination program, covering four cohorts annually (women 11, 14, 17 and 24 years), has been implemented in Basilicata since 2007. This study evaluated vaccine and non-vaccine HPV prevalence 5-7 years post-vaccination program implementation in vaccinated and unvaccinated women.

**Methods:**

This population-based, cross-sectional study was conducted in the public screening centers of the Local Health Unit in Matera between 2012 and 2014. Cervical samples were obtained for Pap and HPV testing (HC2, LiPA Extra® assay) and participants completed a sociodemographic and behavioral questionnaire. Detailed HPV vaccination status was retrieved from the official HPV vaccine registry. HPV prevalence was described overall, by type and vaccination status. The association between HPV type-detection and risk/protective factors was studied. Direct vaccine protection (qHPV vaccine effectiveness [VE]), cross-protection, and type-replacement were evaluated in cohorts eligible for vaccination, by analyzing HPV prevalence of vaccine and non-vaccine types according to vaccination status.

**Results:**

Overall, 2793 women (18-50 years) were included, 1314 of them having been in birth cohorts eligible for the HPV vaccination program (18- to 30-year-old women at enrolment). Among the latter, qHPV vaccine uptake was 59% (at least one dose), with 94% completing the schedule; standardized qHPV type prevalence was 0.6% in vaccinated versus 5.5% in unvaccinated women (*P* <0.001); adjusted VE against vaccine type infections was 90% (95% CI: 73%-96%) for all fully vaccinated women and 100% (95% CI not calculable) in women vaccinated before sexual debut. No statistically significant difference in overall high-risk HPV, high-risk non-vaccine HPV, or any single non-vaccine type prevalence was observed between vaccinated and unvaccinated women.

**Conclusions:**

These results, conducted in a post-vaccine era, suggest a high qHPV VE and that a well-implemented catch-up vaccination program may be efficient in reducing vaccine-type infections in a real-world setting. No cross-protective effect or evidence of type-replacement was observed a few years after HPV vaccine introduction.

**Electronic supplementary material:**

The online version of this article (10.1186/s12879-018-2945-8) contains supplementary material, which is available to authorized users.

## Background

Genital human papillomavirus (HPV) infection is the most common sexually transmitted viral infection worldwide, with approximately 75% of sexually active men and women exposed to HPV during their lives [1].

HPV types that can infect the genital tract can be categorized as high risk (HR) or low risk (LR) according to the degree of risk associated with the development of cervical cancer. Infection with HR-HPV is found in virtually all cases of cervical cancer and is considered a necessary cause of invasive cervical cancer [2]. Among the 12 HPV types considered carcinogenic, HPV16 and HPV18 are the most aggressively oncogenic [3], and are estimated to cause about 70% of invasive cervical cancer cases [4]. Other oncogenic HPV types, mainly HPV 31, 33, 45, 52, and 58, are estimated to be responsible for another 18% of cervical cancers [4, 5].

In Italy, cervical cancer is the 15th most frequent cancer overall, and the third most frequent among women aged 15-44 years [6]. Every year, about 3000 women are newly diagnosed with cervical cancer, of whom 1000 die from the disease. The overall HPV prevalence for Italy in women with normal cytology has been estimated at 9.7% (95% confidence interval [CI]: 9.4-10) [6].

Large randomized controlled trials (RCTs) have shown HPV vaccines to be well tolerated and highly efficacious against vaccine-type persistent HPV infection and precancerous cervical lesions (vaccine efficacy 93% to 100%) [7, 8]. HPV immunization programs are now implemented in many countries, although the methods of delivery, uptake, and monitoring systems vary [9], as does the choice of vaccine [10]. A national HPV vaccination program had been started in all 21 regions of Italy by 2008.

In Basilicata, a region in southern Italy, an organized vaccination program was launched in 2007 with the prophylactic quadrivalent HPV (qHPV) vaccine Gardasil® (Merck Sharp & Dohme Corp, Whitehouse Station, NJ). A unique multiple-cohort vaccine implementation strategy was used, inviting yearly four birth cohorts (girls and women during the 11th, 14th, 17th, and 24th year of age) to be vaccinated free of charge in the Local Health Unit (LHU) of Matera. The first cohorts were born in 1996, 1993, 1990, and 1983, respectively. In addition, HPV vaccinations given through this organized program are systematically reported into a computerized vaccination registry. Cervical screening targeting women aged between 25 and 64 years is also well organized in Basilicata, and the adherence rate was on average 57% for 2013 to 2014 (Ronco G, personal communication).

This cross-sectional population-based study aimed to assess vaccine and non-vaccine HPV prevalence 5-7 years post-vaccination program implementation in vaccinated and unvaccinated women.

## Methods

### Study participants and setting

All 15 public screening centers managed by the LHU of Matera took part in the study. These centers used computerized HPV vaccine and screening registries. Between 21 May 2012 and 14 February 2014, all women aged 25-50 years participating in the Basilicata cervical cancer screening program were asked to take part in the study. Women aged 18-24 years, who were not age-eligible for the screening program, were identified from municipality registries and all were also invited by individual letters to participate in the study. Those not yet sexually active, hysterectomized women, and women who had undergone colposcopy in the 24 months prior to the enrolment visit were not eligible for enrolment. Eligible women were enrolled after signing an informed consent form.

At the enrolment visit, two cervical samples (one each for Pap and HPV testing) were obtained, and participants completed self-administered sociodemographic and behavioral questionnaires. HPV vaccination history, HPV and cytological data, sociodemographic and behavioral data, including sexual habits and age at first sexual intercourse, were recorded. Detailed individual HPV vaccination status (including date of each vaccination and number of doses received) was also retrieved from the official computerized HPV vaccine registry of the LHU of Matera. This information was used for the statistical analysis. HPV analyses and genotyping were centralized in an accredited laboratory at the Cancer Prevention and Research Institute (ISPO). Quality control of HPV testing, cytology, genotyping, and data input procedures were implemented throughout the study, which was conducted in accordance with the Good Epidemiological Practice guidelines and the principles of the Declaration of Helsinki, and was approved by the Matera LHU ethics committee.

### Laboratory methods

#### HC2 assay

HPV detection was performed by Hybrid Capture® 2 (HC2; QIAGEN, Hilden, Germany) using HR-HC2 (to detect HPV 16, 18, 31, 33, 35, 39, 45, 51, 52, 56, 58, 59, and 68) and LR-HC2 (to detect HPV 6, 11, 42, 43, and 44) probes with a semi-automated procedure (Rapid Capture® system, QIAGEN). As recommended by the manufacturer, samples with a relative light units (RLU) to cutoff value (CO) ratio ≥1.0 were classified as HPV positive.

#### HPV genotyping

Genotyping was performed for all HC2 positive samples (HR and/or LR positive; RLU/CO ratio ≥1), all “borderline” samples (RLU/CO ratio <1 and ≥0.5), and 10% of HC2 negative samples (RLU/CO ratio <0.5) chosen randomly. After DNA extraction (QIAamp DNA Mini Kit, QIAGEN), samples were amplified by INNO-LiPA® HPV Genotyping Extra Amp (Innogenetics, Ghent, Belgium) followed by detection by reverse hybridization with HPV type-specific probes for 28 different HPV types: HPV 16, 18, 31, 33, 35, 39, 45, 51, 52, 56, 58, 59 (classified by International Agency for Research on Cancer [IARC] as “carcinogenic to humans”); HPV 26, 53, 66, 68, 70, 73, 82 (classified as “possibly carcinogenic to humans”) and HPV 6, 11, 40, 43, 44, 54, 69, 71, 74 (not classifiable as to its carcinogenicity to humans). Results were interpreted using LiRAS® for LiPA HPV software (Fujirebio Europe, Ghent, Belgium) followed by manual verification. When the INNO-LiPA test did not exclude the presence of HPV 39, 52, 68, or 56 co-infection, an additional type-specific polymerase chain reaction (PCR) was performed. PCR with specific HPV42 primers was performed on LR-HC2 positive or borderline samples (HR-HPV HC2 ratio <1.0, ≥0.5) that were INNO-LiPA negative, as HC2 may detect HPV42 that cannot be detected by INNO-LiPA.

### Statistical methods

Sociodemographic characteristics (birth place, marital status, educational level, occupational status) collected in the self-administered questionnaire were compared with data from the Italian National Institute of Statistics (ISTAT; www.demo.istat.it) to assess whether the study population was representative of the general female population of Basilicata [11].

Crude, age-stratified, and age-standardized prevalence (with related 95% CI) were estimated overall, as well as for HR types, HR non-vaccine types, HR vaccine types, LR types, LR non-vaccine types, LR vaccine types, all vaccine types, and all non-vaccine types, as well as by HPV type. The female Italian National Population (1 January 2012 census) was used as the standard population. For women born in cohorts eligible for the HPV vaccination program (1983 to 1996, corresponding to women aged 18-30 years within the study recruitment period), overall and type-specific data were stratified by vaccination status.

Information on vaccination status, derived from the official computerized Matera LHU vaccine registry, was defined at the date of informed consent. Women who received the first vaccine dose before the date of informed consent were considered as vaccinated (women who received the first vaccine dose at the time of informed consent were considered as unvaccinated). A sensitivity analysis was performed considering as vaccinated only women with a complete vaccination schedule with all three doses administered before the consent date. Age at first sexual intercourse was self-reported and used to identify women who had been vaccinated before sexual debut.

Direct vaccine protection (effect on qHPV vaccine types), cross-protection (effect on types closely related to vaccine types; i.e., HPV 31, 33, and 45), and potential type-replacement (effect on HR non-vaccine types; HR non-16,18), HR non-vaccine non-cross-protective types (i.e., HR non-16, 18, 31, and HR-non 16, 18, 31, 33, 45), and each single HR non-vaccine HPV types (provided N >10) were evaluated in cohorts eligible for vaccination, by analyzing HPV prevalence of vaccine and non-vaccine types according to vaccination status. Comparison of standardized HPV prevalence in vaccinated and unvaccinated participants was obtained using a Poisson regression model adjusted for 5-year age groups. The difference between HPV prevalence by 5-year age groups was tested using the chi-squared test. Logistic regression was used to evaluate the relationship between cervical HPV infection and potential risk or protective factors, including vaccination. A univariate analysis was used to identify associations between HPV infection and each potential risk/protective factor. Factors for which an association with HPV infection was found (*P* ≤0.1) were included in the multivariate analysis. In the final multivariate logistic regression model, vaccine effectiveness (VE) of a full vaccination schedule was evaluated for the prevention of qHPV vaccine type detection as the complement of the adjusted odds ratio (aOR) of the vaccination status (VE = (1 – aOR)*100), in cohorts eligible to the vaccination program (18- to 30-year-old women). Similar analyses compared HPV prevalence in women vaccinated before sexual debut and unvaccinated women. Due to the low number of women vaccinated before sexual debut in the 25- to 30-year group, these comparisons were restricted to those aged 18-24 years. Statistical analyses were performed using STATA® (version 11 or 12) software (StataCorp LP, College Station, Texas, US).

## Results

### Description of study population

All 8349 women aged 18-24 years living in the Matera area were invited to participate in the study. Among them, only 906 presented themselves to screening centers, and of those, 895 accepted and satisfied the eligibility criteria for enrolment (i.e., 10.7% of women initially contacted). The proportion of women aged 25-50 years attending for cervical cancer screening who agreed to participate in the study was >98% (1909/1941). Overall, 2793 women were included in the statistical analysis (11 enrolled women were excluded either because sample dates were inconsistent or because samples had not been sent for testing), 894 and 1899 in the 18-24 years and 25- to 50-year groups, respectively.

Study population characteristics are described in Additional file 1: Table 1a and b. The demographic profile of the study population was generally representative of the overall Italian population, and comparison with ISTAT data revealed a slightly higher than expected proportion of women born outside Italy among participants in both age groups (18-24 years and 25-50 years), especially in the younger group (study: 8.4%; ISTAT: 4.1%). The proportion of married women in the 25- to 50-year group (study: 68.1%) was similar to that expected (ISTAT: 67.6%), but the proportion was higher in the younger age group (study: 7.9%; ISTAT: 4.7%). In the 25- to 50-year group, the proportion of participants with a high school diploma or higher was 11% higher than expected (study: 76.3%; ISTAT: 65.5%), whereas in the younger age group the proportion was quite similar (study: 84.6%; ISTAT: 87.6%).

**Table 1:** HPV vaccination status by age group

	N° Enrolled Women	Unvaccinated	Vaccinated
Age at enrolment (years)^a^		*N*° (%)	*N*° (%)
18-24	894	297 (33.2%)	597 (66.8%)
25-30	420	242 (57.6%)	178 (42.4%)
31-35	315	308 (97.8%)	7 (2.2%)
36-40	435	435 (100.0)	0 (0.0%)
41-45	396	395 (99.7%)	1 (0.3%)
46-50	333	333 (100.0%)	0 (0.0%)
Age at enrolment (years)^b^		N° (%)	N° (%)
18-30	1314	539 (41.0%)	775 (59.0%)
31-50	1479	1471 (99.5%)	8 (0.5%)
Total	2793	2010 (72.0%)	783 (28.0%)

^a^5-year age groups

^b^Age group eligible and not eligible for vaccination

Table 1 shows age distribution by vaccination status. Among women included in the analysis, 1314 were in birth cohorts eligible for the HPV vaccination program since its introduction (18- to 30-year-old women in study). The proportion of vaccinated women differed significantly by age class (*P* <0.001): 67% in the 18- to 24-year group, and 42% in the 25- to 30-year group (overall 59% in those 18-30 years), whereas it was near zero among women older than 30 years.

Among vaccinated women, 38.4% (301/783) had received their first dose before sexual debut. This value reached to ≈50% in the 18- to 24-year group (296/597) and then dropped sharply in the older age group (2.8% for those 25-30 years and 0% for those 31-55 years).

Almost all women (94%) who had received at least one dose had gone on to complete the full vaccination schedule of three doses. Schedule completion was higher in women who had been vaccinated before sexual debut, with 97% receiving all three doses.

Within the 18- to 30-year subgroup, the demographic profiles of vaccinated and unvaccinated women differed for some parameters: vaccinated women were on average younger than unvaccinated women, more frequently born in Italy (98% vs 84%), single (92% vs 71%), with a higher educational level (84% vs 72%), and more frequently unemployed (see Table 2). However, the two groups were comparable for the main risk factors for HPV infection: the number of sexual partners both in the last 6 months and over their lifetime (84% of both vaccinated and unvaccinated women had one sexual partner in the last 6 months and ≈61% of both vaccinated and unvaccinated women had more than one lifetime partner), and they had similar smoking habits (*P* =0.268) (see Table 3).

**Table 2:** Comparison between vaccinated and unvaccinated women (aged 18-30 years) by sociodemographic characteristics (*N* =1314)

	Unvaccinated		Vaccinated		*P*-value^a^
	*n* = 539		*n* = 775		
	*n*	%	*n*	%	
Age at enrolment					<0.001
18-24 y	297	55.1	597	77.0	
25-30 y	242	44.9	178	23.0	
Country of birth					<0.001
Italy	455	84.4	759	97.9	
Other	83	15.4	14	1.8	
Unknown	1	0.2	2	0.3	
Marital status					<0.001
Single	383	71.1	712	91.9	
Married	138	25.6	54	7.0	
Divorced/separated	10	1.9	4	0.5	
Unknown	8	1.5	5	0.6	
Educational level					<0.001
Secondary school or lower	135	25.0	110	14.2	
High school diploma or higher	386	71.6	648	83.6	
Other	15	2.8	13	1.7	
Unknown	3	0.6	4	0.5	
Occupational status					0.014
Employed	167	31.0	189	24.4	
Not working^b^	369	68.5	583	75.2	
Other	1	0.2	0	0.0	
Unknown	2	0.4	3	0.4	

^a^The *P*-values were calculated excluding data from the “missing” category

^b^Includes those looking for a job, housewives, those at high school, university students, and the unemployed

**Table 3:** Comparison between vaccinated and unvaccinated women (aged 18-30 years) by behavioral characteristics (*N* =1314)

	Unvaccinated n = 539		Vaccinatedn = 775		*P*-value^a^
	*n*	%	*n*	%	
Smoking status					0.268
Yes	210	39.0	312	40.3	
No, I quit	77	14.3	88	11.4	
Never	240	44.5	362	46.7	
Unknown	12	2.2	13	1.7	
Ever been pregnant					<0.001
Yes	153	28.4	59	7.6	
No	376	69.8	700	90.3	
Unknown	10	1.9	16	2.1	
Use of condom					0.001
Current use (regardless of the past)	272	50.5	455	58.7	
Only past use	6	1.1	19	2.5	
Never used	138	25.6	142	18.3	
Unknown	123	22.8	159	20.5	
Number of sexual partners in past 6 months					0.768
None	24	4.5	44	5.7	
1	455	84.4	655	84.5	
2-4	44	8.2	69	8.9	
5+	2	0.4	2	0.3	
Unknown	14	2.6	5	0.6	
Number of lifetime sexual partners					0.270
1	204	37.8	288	37.2	
2-4	237	44.0	372	48.0	
5+	81	15.0	97	12.5	
Unknown	17	3.2	18	2.3	

^a^The *P*-values were calculated excluding data from the “missing” category

### Overall HPV prevalence and type distribution

The overall standardized HPV prevalence in the study population (women aged 18-50 years) was 9.5% for all types, 7.6% for HR-HPV types, and 3.2% for LR-HPV types (Additional file 1: Table S2). Overall, HPV prevalence was higher in younger than older age groups, with a significant trend of decreasing prevalence with increasing age; this trend was similar for all HR types and all LR types.

The overall standardized prevalence of non-vaccine HPV types was 8.0% (4.9% for HR non-vaccine types) and 2.4% for the four qHPV vaccine types combined (HPV 6, 11, 16, and 18) (2.3% for HR-qHPV vaccine types) (Additional file 1: Table S3). The decreasing trend with increasing age was not observed for qHPV vaccine types. Instead, the prevalence of qHPV vaccine types was lower in women age-eligible for vaccination (2.5% for women aged 18-24 years, and 3.1% for those aged 25-30 years for all four HPV vaccine types) compared with women aged 31-35 years (5.1%) (*P* =0.027).

### HPV prevalence by vaccination status – restricted to cohorts eligible for vaccination program (18- to 30-year group)

#### Overall HPV prevalence

**Fig. 1:**
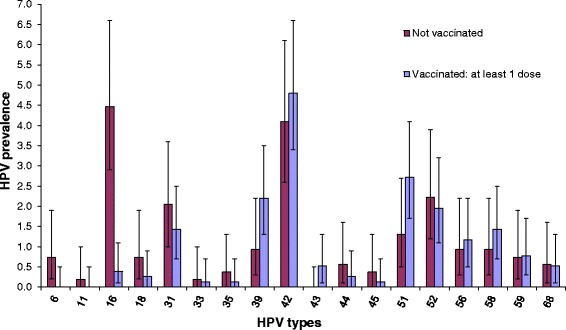
HPV type prevalence by vaccination status (women aged 18–30 y)

Among the cohorts eligible for the vaccination program, the most frequent HPV type in unvaccinated women was HPV16, followed by types 42, 52, 31, and 51. In vaccinated women, HPV42 was the most frequent type, followed by types 51, 39, and 52, with the same prevalence as types 31 and 58 (Fig. 1). HPV 16 was the only HR type for which a statistically significant difference was observed between vaccinated and unvaccinated women (Fig. 1).

No statistically significant differences were observed between unvaccinated and vaccinated women in the overall standardized HPV prevalence (17.1% vs 15.8%, *P* =0.39), HR-HPV type prevalence (13.9% vs 12.1%, *P* =0.21), and LR-HPV type prevalence (6.0% vs 6.1%, *P* =0.96) (Table 4). HR-HPV prevalence was significantly higher in unvaccinated women compared with women vaccinated before sexual debut (16.6% vs 9.5%, *P* =0.01 in the 18- to 24-year old group) (Table 5). This difference was maintained after adjustment for age at enrolment, marital status, smoking status, number of sexual partners (within the past 6 months and lifetime), and sexually transmitted disease (past or present), although this was no longer statistically significant (aOR =0.62, *P* =0.09) (Table 5).

**Table 4:** HPV standardized prevalence (%) stratified by vaccination status and adjusted odds ratio evaluating association between HPV prevalence and vaccination status (women 18-30 years)

	HPV Prevalence % (N°)			Adjusted^a^ OR Vaccinated vs Unvaccinated	
HPV types	Unvaccinated (*n* = 537)	Vaccinated (*n* = 771)	*P*-value	aOR (95% CI)	*P*-value
All types	17.1 (*n* 93)	15.8 (*n* 124)	0.39	0.89 (0.64-1.24)	0.495
HR types	13.9 (*n* 76)	12.1 (*n* 94)	0.21	0.79 (0.55-1.13)	0.203
LR types	6.0 (*n* 33)	6.1 (*n* 51)	0.96	1.00 (0.61-1.63)	0.989
Vaccine types	5.5 (*n* 30)	0.6 (*n* 5)	<0.001	0.10 (0.04-0.27)	<0.001
HR vaccine types (16,18)	5.2 (*n* 28)	0.6 (*n* 5)	<0.001	0.11 (0.04-0.30)	<0.001
LR vaccine types (6,11)	0.7 (*n* 4)	0.0 (*n* 0)	0.02	NE	-
Non-vaccine types^b^	14.3 (*n* 78)	15.3 (*n* 120)	0.88	1.08 (0.76-1.52)	0.681
HPV 31	2.1 (*n* 11)	1.3 (*n* 11)	0.58	0.89 (0.36-2.20)	0.795
HPV 39	0.9 (*n* 5)	1.8 (*n* 17)	0.22	2.05 (0.69-6.05)	0.196
HPV 51	1.3 (*n* 7)	2.9 (*n* 21)	0.08	2.40 (0.94-6.13)	0.067
HPV 52	2.2 (*n* 12)	2.1 (*n* 15)	0.60	0.75 (0.33-1.70)	0.495
HPV 56	0.9 (*n* 5)	1.0 (*n* 9)	0.94	1.17 (0.36-3.82)	0.790
HPV 58	0.9 (*n* 5)	1.3 (*n* 11)	0.58	1.16 (0.39-3.46)	0.791
HPV 59	0.7 (*n* 4)	0.5 (*n* 6)	0.77	0.89 (0.23-3.48)	0.870
HPV 31, 33, 45	2.7 (*n* 14)	1.5 (*n* 13)	0.30	0.95 (0.40-2.27)	0.908
HR-HPV no 16, 18	8.6 (*n* 47)	8.8 (*n* 73)	0.97	1.03 (0.68-1.58)	0.879
HR-HPV no 16, 18, 31	6.9 (*n* 38)	8.3 (*n* 68)	0.56	1.14 (0.72-1.79)	0.574
HR-HPV no 16, 18, 31, 33, 45	6.3 (*n* 35)	8.2 (*n* 67)	0.35	1.24 (0.78-1.97)	0.356
LR non-vaccine types	4.6 (*n* 25)	5.2 (*n* 43)	0.66	1.17 (0.68-2.02)	0.576

*HR* high risk, *LR* low risk, *OR* odds ratio, *aOR* adjusted OR, *CI* confidence interval, *NE* not estimable

^a^Association adjusted for the following variables: marital status, smoking status, number of sexual partners in the past 6 months, number of lifetime sexual partners, and sexually transmitted diseases

^b^HPV 33, 35, 43, 45, and 68 were tested but as the overall number of positive sample was <10 for each of these types, HPV prevalence was not stratified by vaccination status

**Table 5:** HPV standardized prevalence (%) stratified by vaccination status and adjusted odds ratio evaluating association between HPV prevalence and vaccination status (women 18-24 years)

	HPV Prevalence % (N°)				Adjusted^a^ OR Vaccinated Before Sexual Debut vs Unvaccinated	
HPV types	Unvaccinated (n = 295)	Vaccinated before sexual debut (*n* 295)	Vaccinated after sexual debut (*n* 294)	*P*-value^b^	aOR (95% CI)	*P*-value
All types	20.0 (*n* 59)	14.6 (*n* 43)	18.4 (*n* 54)	0.08	0.89 (0.55-1.43)	0.630
HR types	16.6 (*n* 49)	9.5 (*n* 28)	15.3 (*n* 45)	0.01	0.62 (0.36-1.07)	0.088
LR types	7.5 (*n* 22)	6.8 (*n* 20)	7.5 (*n* 22)	0.74	1.19 (0.60-2.36)	0.613
Vaccine types	6.1 (*n* 18)	0.0 (*n* 0)	1.4 (*n* 4)	<0.001	0.00 (NE)	-
HR vaccine types (16,18)	5.8 (*n* 17)	0.0 (*n* 0)	1.4 (*n* 4)	<0.001	0.00 (NE)	-
LR vaccine types (6,11)	1.0 (*n* 3)	0.0 (*n* 0)	0.0 (*n* 0)	0.08	0.00 (NE)	-
Non-vaccine types^c^	16.9 (*n* 50)	13.9 (*n* 41)	18.0 (*n* 53)	0.31	1.04 (0.63-1.70)	0.888
HPV 31	1.0 (*n* 3)	0.7 (*n* 2)	2.4 (*n* 7)	0.69	0.73 (0.12-4.57)	0.740
HPV 39	1.7 (*n* 5)	1.7 (*n* 5)	3.4 (*n* 10)	1.00	1.37 (0.36-5.26)	0.643
HPV 51	1.4 (*n* 4)	1.0 (*n* 3)	4.1 (*n* 12)	0.66	1.02 (0.20-5.05)	0.984
HPV 52	3.1 (*n* 9)	1.7 (*n* 5)	2.0 (*n* 6)	0.27	0.77 (0.24-2.50)	0.663
HPV 56	1.4 (*n* 4)	0.7 (*n* 2)	2.0 (*n* 6)	0.40	0.63 (0.11-3.78)	0.615
HPV 58	1.4 (*n* 4)	2.0 (*n* 6)	1.0 (*n* 3)	0.57	1.83 (0.47-7.12)	0.385
HPV 59	1.0 (*n* 3)	0.7 (*n* 2)	1.4 (*n* 4)	0.69	0.78 (0.13-4.82)	0.790
HPV 31, 33, 45	2.0 (*n* 6)	0.7 (*n* 2)	3.1 (*n* 9)	0.17	0.73 (0.12-4.24)	0.722
HR-HPV no 16, 18	10.2 (*n* 30)	7.8 (*n* 23)	12.6 (*n* 37)	0.31	0.97 (0.52-1.80)	0.917
HR-HPV no 16, 18, 31	9.5 (*n* 28)	7.5 (*n* 22)	11.2 (*n* 33)	0.38	0.97 (0.52-1.83)	0.934
HR-HPV no 16, 18, 31, 33, 45	8.5 (*n* 25)	7.5 (*n* 22)	10.9 (*n* 32)	0.65	1.09 (0.57-2.08)	0.786
LR non-vaccine types	5.4 (*n* 16)	5.1 (*n* 15)	6.8 (*n* 20)	0.87	1.26 (0.58-2.76)	0.560

types, HPV prevalence was not stratified by vaccination status

*HR* high risk, *LR* low risk, *OR* odds ratio, *aOR* adjusted OR, *CI* confidence interval, *NE* not estimable

^a^Association adjusted for the following variables: marital status, smoking status, number of sexual partners in the past 6 months, number of lifetime sexual partners, and sexually transmitted diseases

^b^Comparison between unvaccinated and vaccinated before sexual debut. Note: nine cases of vaccinated women for whom there was no information about age at first sexual intercourse were excluded from the analysis

^c^HPV33, 35, 43, 45, and 68 were tested but as the overall number of positive sample was <10 for each of these

#### qHPV vaccine type prevalence and vaccine effectiveness

The standardized prevalence of qHPV vaccine types (16, 18, 6, 11) was significantly higher in unvaccinated than in vaccinated women (5.5% vs 0.6%, *P* <0.001) (Table 4). This was true for both HR-qHPV vaccine types (5.2% vs 0.6%, *P* <0.001) and LR-qHPV vaccine types (0.7% vs 0.0%, *P* =0.02). The corresponding adjusted VE against vaccine type infections was 90% (95% CI: 73%-96%). No qHPV vaccine type infections were detected among women vaccinated before sexual debut (Table 5), corresponding to a VE of 100% against vaccine type infections in this population compared with unvaccinated women in the same age group (18-24 years).

#### Non-vaccine type prevalence: cross-protection and type-replacement

Even though we observed a higher prevalence point estimate for HPV 31, and HPV 31, 33, and 45 combined in unvaccinated versus vaccinated women, these differences were not statistically significant (2.1% vs 1.3%, *P* =0.58, and 2.7% vs 1.5%, *P* =0.30, respectively; Table 4). No statistically significant differences in HPV prevalence between unvaccinated and vaccinated women were observed in these cohorts for HR non-vaccine HPV types; HR non-16, 18, 31 HPV types; HR non-16, 18, 31, 33, 45 HPV types, or for any single non-vaccine HPV type (Table 4).

Similar results were observed when women vaccinated before sexual debut were compared with unvaccinated women of the same age-group and after adjustment for confounding (Tables 4 and 5). All analyses were repeated considering as vaccinated only women who completed all three doses before the consent date; similar results were found (data not shown).

## Discussion

Our study is one of the largest in regard to the number of women enrolled in a post-vaccine era (1314 women age-eligible for the vaccination program were included). Due to this large sample size, the wide age range of women targeted by the vaccination program in Basilicata (11- to 24-year-old women each year), as well as the very good overall adherence to the vaccination program (about 59% vaccine uptake among all age ranges; 94% adherence to a full vaccination schedule), this region provides a unique setting to evaluate the real-life effect of a well-implemented vaccination program on viral HPV circulation, particularly on HPV types under the selective pressure of qHPV vaccine introduction.

This is also the first study analyzing overall HPV and type-specific prevalence in a post-vaccine era in Italy. By performing a comprehensive genotyping assay for surveillance, rather than focusing on HPV 16/18 only, we aimed to gain insights into cross-protection and type-replacement, in addition to vaccine effectiveness, as well as to provide a good baseline to monitor the impact of the possible future introduction of a nonavalent HPV vaccine.

A strength of this study was the high quality of the information gathered on individual vaccination status, which was issued from the official computerized vaccine registry of the LHU of Matera ensuring differentiation between vaccinated and unvaccinated individuals and allowing for a robust vaccine effectiveness evaluation. The collection of sociodemographic and behavioral data, using an anonymous questionnaire, is another strength of this study, as well as facilitated adjusted comparisons between vaccinated and unvaccinated women with several confounding factors for HPV infection in multivariate analysis. In particular, the self-declared age at first sexual intercourse allowed differentiation between women vaccinated before sexual debut, who had the potential to fully benefit from the vaccination, and those who were likely to have been exposed to HPV before vaccination.

One limitation of the study is the low participation rate (10.8%) of women not age-eligible for the organized screening program (aged 18-24 years). This participation rate, lower than that expected based on previous studies in Italy [12, 13], was mainly due to the large proportion of women in this age group who were attending university outside of Matera province for most of the year. This may have limited the number of the youngest study participants, leading to a lower than planned number of participants in cohorts age-eligible for the vaccination program. Thus, the results concerning the overall and type-specific HPV prevalence stratified by vaccination status, particularly the evaluation of type-replacement, must be interpreted with caution, as the study was not sufficiently powered to analyze the impact of vaccination on single HPV types. With a sample size equal to 1314 women (41% unvaccinated and 59% vaccinated) and a power of 80%, we were able to show a statistically significant reduction in prevalence of 2.8% for HPV 16/18 and 4.6% for HR-HPV types between unvaccinated and vaccinated women.

Another limitation is that our study was conducted in a post-vaccine era only. In absence of HPV prevalence data collected in a similar manner in a pre-vaccine era (same centers, same region, same analysis method, etc.), we cannot fully assess the impact of the vaccine program introduction. Nevertheless, our data seem overall consistent with HPV prevalence data observed in pre-vaccine eras elsewhere in Italy. For example, the HPV prevalences observed in 2007 in a screened population aged 25-65 years living in central and southern Italy [14] were very close to those observed in our population (18-50 years): 11.9% versus 11.3% for overall HPV; 9.2% versus 8.9% for HR-HPV, and 4.2% versus 4.1% for LR-HPV prevalence, respectively. The HPV prevalence observed among unvaccinated women aged 18-24 years in our study is consistent with figures from an HPV study conducted in southern Italy in 2006 to 2007 [13] and data from studio PREGIO conducted in 2007 to 2009 [12]. Lastly, it is important to underline that in the Basilicata region, the HPV vaccination program was limited only to female birth cohorts (11, 14, 17, and 24 years), and not for the whole population. Thus, also considering that the HPV prevalence data for unvaccinated women in our study were highly comparable to other Italian pre-vaccine studies, a herd immunity effect is unlikely.

Our population had a high prevalence of HPV42. It is important to note that most prevalence studies in Italy did not genotype LR types or the genotype systems used did not detect HPV 42. In the present study, all genotyping analyses were performed by INNO-LiPA Genotyping Extra, which is not able to detect HPV 42, one of the LR-HPV HC2 target types. Consequently, we evaluated HPV 42 prevalence by type-specific PCR, for all LR-HC2 positive samples that were negative for specific HPV genotypes as detected by INNO-LiPA. This may explain why the prevalence of HPV 42 is so high in our study.

The high prevalence of HPV infection in the youngest age groups and decreasing HPV prevalence with increasing age was expected from HPV natural history [15–18]. Still, it was interesting to note that this trend was not observed for qHPV vaccine types. Indeed, the prevalence of qHPV vaccine types was statistically significantly lower in women age-eligible for vaccination (2.5% for women aged 18-24 years; 3.1% for those aged 25-30 years) compared with the prevalence observed in the 31- to 35-year group (5.1%), indicating a probable impact of this vaccination program at the population level.

In our study, VE against qHPV vaccine types was 90% considering all vaccinated women (18-30 years), regardless of sexual exposure at the time of vaccination. These figures are close to those observed by Tabrizi [19], with a VE of 73% against qHPV vaccine-types among women aged 18-24 years (89% after adjusting for age and use of hormonal contraceptives), and more recently by Markowitz et al. (VE of 89% against qHPV vaccine types in women aged 14-24 years) [20]. HPV vaccination is not therapeutic [21], and women vaccinated after sexual debut may have already been exposed to vaccine HPV types [22]. In the present study, a substantial proportion of women (60%) were sexually active at vaccination. However, the overall VE was high, suggesting that a well-implemented catch-up vaccination program may be successful. No qHPV vaccine type infections were detected in women vaccinated before sexual debut, among which almost all (97%) had completed the full vaccination schedule. The high VE of 100% in this population confirms that the full benefit of vaccination is obtained when women are vaccinated with a complete vaccination schedule before sexual debut. These data also confirm the results observed in qHPV vaccine RCTs [7, 23] in a real-life setting. Due to the low number of women who had not completed a full vaccination schedule in our study (19 women received only one dose, 29 women received only two doses), we did not directly assess the VE of an incomplete vaccination schedule.

Although not statistically significant, lower HPV prevalence point estimates of HPV 31, 33, and 45 were observed in vaccinated versus unvaccinated women, suggesting that the qHPV vaccine might provide some degree of cross-protection against non-vaccine HPV types. This seems in line with evidence from other clinical [24–26] and observational studies [26], even though the limited power of the current study precludes firm conclusions on this point.

Vaccination is expected to reduce the prevalence of HPV vaccine types, but there is a theoretical concern that vaccine introduction may affect the distribution of other oncogenic types and induce type-replacement [27]. Similar to other observational studies [20, 28–30], our data provide no evidence of type-replacement a few years after HPV vaccine introduction. In their meta-analysis. Drolet et al. [29] found no statistically significant difference in the prevalence of HR-non-vaccine types between pre- and post-vaccination periods in any of the age groups studied (13-19 years and 20-24 years). In women aged 20-24 years, a small, but not statistically significant, increase in non-vaccine HR-HPV types (relative risk: 1.09, 95% CI: 0.98-1.22) was found to be negatively associated with increasing vaccination coverage (*P* =0.03) [29]. A recent meta-analysis by Mesher et al. [31] shows a significant increase in some HR non-vaccine types (HPV 39, HPV 52) in the post-vaccine era. These results, as well as the HPV types found to be significantly increased, are inconsistent between age groups, vaccine type (bivalent vs quadrivalent), and according to the methodological quality of studies, suggests random fluctuation. When only studies with a low potential for bias were considered, no statistically significant difference was observed. Having considered the potential limitations and uncertainties of this approach to evaluate type-replacement, Mesher et al. concluded that their meta-analysis provided no clear evidence for type-replacement [31]. This finding needs further exploration.

“Unmasking,” a potential diagnostic artifact of broad-spectrum assays, may give rise to an apparent increase in non-vaccine types following vaccination [27, 32], and may partially explain small observed increases. In the presence of high concentration types, broad-spectrum assays can miss types that are present in much lower concentrations. As HPV 16/18 are usually the most prevalent types, other types are less likely to be detected when present in the same samples. In vaccinated subjects, non-vaccine types that may not have been detected in the absence of vaccination due to the presence of HPV 16/18, may be “unmasked” by vaccination and thus be detected more broadly in vaccinated versus unvaccinated subjects.

“Unmasking” should be differentiated from type-replacement. It is equally important to evaluate the test used and ensure that it performs adequately. The World Health Organization HPV LabNet provides blinded “proficiency panels” designed to evaluate whether assays can detect a monoinfection equally well in the presence of other HPV types. Comparison of results from more than 100 laboratories worldwide that have used a variety of HPV assays has shown that underestimation of some HPV types, when other types are present in the same sample, is a problem for some assays [33, 34]. In this regard, continuing monitoring to ensure the adequate performance of assays used for surveillance is important.

The results of our study will be an important basis for the evaluation of future changes in the epidemiology of HPV infection in Italy, particularly in view of the upcoming 9-valent HPV vaccine that protects against five additional oncogenic HPV types [35]. Ongoing monitoring of the incidence of cervical pre-cancerous lesions and cancers, as well as other HPV-related lesions, such as anal and oropharyngeal cancers, will also be essential to understand the overall cost-effectiveness and population level benefit of HPV vaccines on cancer prevention. This study provides a preliminary evaluation of the potential effect of the qHPV vaccination program in Basilicata, showing that it significantly reduces the prevalence of qHPV vaccine types in vaccinated populations in a real-life setting compared with the prevalence in unvaccinated women.

## Conclusion

The results of this study, conducted shortly after the implementation of the qHPV vaccination program in Basilicata, provide insight into the real-life population effect of HPV vaccination programs in those 18-50 years. They show a high VE of the qHPV vaccine on vaccine type prevalence, and found no evidence of type-replacement. This effectiveness is expected to be even more important in young girls who are HPV naive at vaccination, and will be examined in the routine cohort of 12-/13-year-olds, in whom vaccine uptake is higher and who will attend their first cervical screening visit in 2021.

## Additional file

Additional file 1: Table S1 A. Description of the sociodemographic characteristics of the study population (N =2793). B. Description of the behavioral characteristics of the study population (N =2793). **Table S2.** Age-stratified, crude, and standardized HPV prevalence (%). **Table S3.** Age-stratified, crude, and standardized HPV prevalence (%) by vaccine and non-vaccine types. (DOCX 23 kb)

## Abbreviations

aOR: Adjusted odds ratio
CI: Confidence interval
CO: Cutoff value
HC2: Hybrid Capture® 2
HPV: Human papillomavirus
HR: High risk
LHU: Local health unit
LR: Low risk
NE: Not evaluable
PCR: Polymerase chain reaction
qHPV: Quadrivalent HPV
RCTs: Randomized controlled trials
RLU: Relative light units
VE: Vaccine effectiveness
