# Supplementary material for: Monitoring vaccine and non-vaccine HPV type prevalence in the post-vaccination era in women living in the Basilicata region, Italy
Source: BMC Infect Dis. 2018 Jan 15;18:38. doi: 10.1186/s12879-018-2945-8 (PMC5769466; doi:10.1186/s12879-018-2945-8)
Supplement: Additional file 1: Table S1 — A. Description of the sociodemographic characteristics of the study population (N =2793). B. Description of the behavioral characteristics of the study population (N =2793). Table S2. Age-stratified, crude, and standardized HPV prevalence (%). Table S3. Age-stratified, crude, and standardized HPV prevalence (%) by vaccine and non-vaccine types. (DOCX 23 kb) [file 12879_2018_2945_MOESM1_ESM.docx]

Additional file 1

Table S1A

Description of the sociodemographic characteristics of the study population (N = 2793)

| **Sociodemographic Characteristics** | | |
| --- | --- | --- |
| **Country of birth** | **n** | **%** |
| Italy | 2590 | 92.7 |
| Other | 194 | 6.9 |
| Unknown | 9 | 0.3 |
| **Marital status** | **n** | **%** |
| Single | 1307 | 46.8 |
| Married | 1351 | 48.4 |
| Divorced/separated | 95 | 3.4 |
| Widowed | 15 | 0.5 |
| Unknown | 25 | 0.9 |
| **Educational level** | **n** | **%** |
| Secondary school or lower | 635 | 22.7 |
| High school diploma or higher | 2082 | 74.5 |
| Other | 62 | 2.2 |
| Unknown | 14 | 0.5 |
| **Occupational status** | **n** | **%** |
| Employed | 1098 | 39.3 |
| Not working^a^ | 1678 | 60.1 |
| Other | 6 | 0.2 |
| Unknown | 11 | 0.4 |

^a^Includes those looking for a job, housewives, those at high school, university students, and the unemployed

Table S1B

Description of the behavioral characteristics of the study population (N = 2793)

| **Behavioral Characteristics** | | |
| --- | --- | --- |
| **Smoking status** | **n** | **%** |
| Yes | 852 | 30.5 |
| No, I quit | 404 | 14.5 |
| Never | 1466 | 52.5 |
| Unknown | 71 | 2.5 |
| **Ever used contraception** | **n** | **%** |
| Yes | 2074 | 74.3 |
| No | 692 | 24.8 |
| Unknown | 27 | 1.0 |
| **Age at first sexual intercourse, y** | **n** | **%** |
| ≤11 | 1 | 0.0 |
| 12-14 | 87 | 3.1 |
| 15-17 | 1077 | 38.6 |
| 18-24 | 1396 | 50.0 |
| >24 | 173 | 6.2 |
| Unknown | 59 | 2.1 |
| **N° sexual partners in past 6 months** | **n** | **%** |
| None | 189 | 6.8 |
| 1 | 2332 | 83.5 |
| 2-4 | 155 | 5.5 |
| 5+ | 14 | 0.5 |
| Unknown | 103 | 3.7 |
| **No. lifetime sexual partners** | **n** | **%** |
| 1 | 1196 | 42.8 |
| 2-4 | 1126 | 40.3 |
| 5+ | 346 | 12.4 |
| Unknown | 125 | 4.5 |

Table S2

Age-stratified crude and standardized HPV prevalence (%)

| **Age Group** | **All Types^a^** | **HR-HPV Types^b^ (16, 18, 31, 33, 35, 39, 45, 51, 52, 56, 58, 59, 68)** | **LR-HPV Types^c^ (6, 11, 42, 43, 44)** |
| --- | --- | --- | --- |
| 18-24 | 17.5 | 13.7 | 7.2 |
| 25-30 | 14.6 | 11.5 | 4.8 |
| 31-35 | 13.1 | 11.2 | 3.2 |
| 36-40 | 5.8 | 3.9 | 2.3 |
| 41-45 | 4.1 | 3.0 | 1.8 |
| 46-50 | 4.5 | 4.2 | 0.6 |
| No. cases/total | 314/2778 | 248/2778 | 113/2778 |
| Crude prevalence, % | 11.3 | 8.9 | 4.1 |
| Standardized prevalence, % | 9.5 | 7.6 | 3.2 |

HR, high-risk; LR, low-risk

^a^Based on HC2 test positivity (HR and/or LR HC2 test positivity): a sample is considered HC2 positive if it contains *at least one* of the following types: HPV 6, 11, 16, 18, 31, 33, 35, 39, 42, 43, 44, 45, 51, 52, 56, 58, 59, 68

^b^Based on HR-HC2 test positivity (at least one type detected)

^c^Based on LR-HC2 test positivity (at least one type detected)

Table S3

Age-stratified, crude, and standardized HPV prevalence (%) by vaccine and non-vaccine types

|  | **qHPV Vaccine Types** | | | **Non-vaccine Types** | | | |
| --- | --- | --- | --- | --- | --- | --- | --- |
| **Age group, y** | **Any vaccine types (6, 11, 16, 18)** | **HR**  **vaccine types (16, 18)** | **LR**  **vaccine types (6, 11)** | **Any**  **non-vaccine types^a^** | **HR non-vaccine types^b^** | **LR non- vaccine types^b^** | **Other non-vaccine HPV types^c^** |
| 18-24 | 2.5 | 2.4 | 0.3 | 16.2 | 10.1 | 5.7 | 6.0 |
| 25-30 | 3.1 | 2.9 | 0.2 | 12.9 | 7.2 | 4.1 | 4.5 |
| 31-35 | 5.1 | 4.5 | 0.6 | 10.9 | 7.0 | 1.6 | 4.5 |
| 36-40 | 1.6 | 1.6 | 0.0 | 3.9 | 2.1 | 1.9 | 1.2 |
| 41-45 | 1.0 | 1.0 | 0.0 | 3.5 | 2.3 | 1.8 | 0.8 |
| 46-50 | 1.8 | 1.8 | 0.0 | 3.0 | 2.1 | 0.3 | 0.3 |
| No. cases/total | 68/2778 | 64/2778 | 6/2778 | 273/2778 | 167/2778 | 89/2778 | 95/2778 |
| Crude Prevalence, % | 2.4 | 2.3 | 0.2 | 9.8 | 6.0 | 3.2 | 3.4 |
| Standardized prevalence, % | 2.4 | 2.3 | 0.2 | 8.0 | 4.9 | 2.5 | 2.7 |

HR, high risk; LR, low risk

^a^Non-vaccine types include: HR non-vaccine types detectable by HC2 (i.e., HPV 31, 33, 35, 39, 45, 51, 52, 56, 58, 59, 68), LR non-vaccine types detectable by HC2 (i.e., HPV 42, 43, 44) and other non-vaccine HPV types (i.e., HPV 26, 40, 53, 54, 66, 69, 70, 71, 73, 74, 82) – at least one type detected

^b^As detected by HC2

^c^Non-detectable by HC2: at least one HPV type among HPV 26, 40, 53, 54, 66, 69, 70, 71, 73, 74, 82
